# 2,4-Dichlorophenol biotransformation using immobilized marine halophilic *Bacillus subtilis* culture and laccase enzyme: application in wastewater treatment

**DOI:** 10.1186/s43141-022-00417-1

**Published:** 2022-09-16

**Authors:** Aida M. Farag, Moustafa Y. El-Naggar, Khaled M. Ghanem

**Affiliations:** 1 Marine Biotechnology and Natural Product Extract, National Institute of Oceanography and Fisheries (NIOF), Alexandria, Egypt; 2 Department of Botany and Microbiology, Faculty of Science, Alexandria University, Alexandria, Egypt; 3 Department of Botany and Microbiology, Faculty of science, Alexandria University, Alexandria, Egypt

**Keywords:** Biodegradation, Chlorophenol, *Bacillus*, Laccase, Immobilization, Wastewaters

## Abstract

**Background:**

2,4-Dichlorophenol (2,4-DCP) is a very toxic aromatic compound for humans and the environment and is highly resistant to degradation. Therefore, it is necessary to develop efficient remediation and cost-effective approaches to this pollutant. Microbial enzymes such as laccases can degrade phenols, but limited information is known about immobilized bacterial laccase and their reuse.

**Methods:**

Immobilization of marine halophilic *Bacillus subtilis* AAK cultures via entrapment and adsorption techniques and degradation of different phenolic compounds by immobilized cells were estimated. Partial purification and immobilization of laccase enzymes were carried out. In addition, the biodegradation of 2,4-DCP and others contaminated by wastewater was investigated.

**Results:**

Immobilization of cells and partially purified laccase enzymes by adsorption into 3% alginate increased 2,4-DCP biotransformation compared with free cells and free enzymes. In addition, the reuse of both the immobilized culture and laccase enzymes was evaluated. The highest removal of 2,4-DCP from pulp and paper wastewater samples inoculated by immobilized cells and the immobilized enzyme was 90% and 95%, respectively, at 50 h and 52 h of incubation, compared to free cells and free enzyme.

**Conclusion:**

The results of this study have revealed the immobilization of a biocatalyst and its laccase enzyme as a promising technique for enhancing the degradation of 2,4-DCP and other toxic phenolic and aromatic compounds. The reuse of the biocatalyst and its laccase enzyme enabled the application of this cost-effective bioremediation strategy.

**Supplementary Information:**

The online version contains supplementary material available at 10.1186/s43141-022-00417-1.

## Background

Chloro-phenolic compounds (CPs) are considered a vital type of xenobiotics due to their extensive use in many important chemical products such as herbicides, insecticides, wood preservatives, dyes, pharmaceuticals, and lubricant additives [1]. The process of chlorination can produce CPs as byproducts which can be used for wastewater disinfection. According to their persistence, CPs affect soil and water (ground and surface) and can be released into the environment for an extended period of time [2]. In addition, they are highly recalcitrant, carcinogenic, and toxic; hence, they are regarded as priority environmental pollutants by the United States Environmental Protection Agency (USEPA) [3]. Different approaches have been used to study the biodegradation of PCs from wastewaters, such as advanced oxidation, photo-catalytic removal, solvent extraction, and adsorption techniques [4]. The accumulation of toxic byproducts and low degradation efficiency are the primary characteristics of these removal techniques. Accordingly, economical and eco-friendly removal methods are preferred for the remediation of polluted toxic aromatic [5]. For process engineers and microbial ecologists, microbial enzymes and cell bioremediation of xenobiotic pollutants remain a significant challenge. Marine microbes are crucial to the biogeochemical cycling of marine ecosystems and marine food webs. Consequently, microbial enzymes produced from marine microorganisms have more advantages than their terrestrial homologs, including genetic manipulation, mass culture ability, biochemical variability, further catalytic activity, lower costs, and sustainability [6]. Recently, most enzymes used in biotechnological and industrial processes are hydrolytic and used to remove aromatic and phenolic compounds from water [7].

Laccases (benzenediol: oxygen oxidoreductase, EC 1.10.3.2) have a high potential for use in a variety of applications. They are multicopper oxidases and catalyze the oxidation of various PCs and some aromatic compounds [8]. Microorganisms can produce laccase enzymes such as fungi [9] and bacteria [10] that are produced by *Bacillus* and *Streptomyces* [11]. In contrast to other remediation techniques, the laccase enzyme does not produce harmful byproducts when removing environmental pollutants.

During the biodegradation process, enzyme and cell immobilization technology can increase microbial biomass and maintain its high activity rate. Furthermore, it has many advantages as the toxicity tolerance is high; the degradative enzyme activity is maintained without the need to extract enzymes from cells. The separation of products is easy, the reaction speed is fast, and the equipment is miniaturized [12].

In biotechnological applications, immobilized enzymes have many advantages: they become highly resistant to environmental conditions, they can be removed easily at the end of the reaction, the product is not contaminated with the enzyme, and enzymes can be reused easily. In addition, product formation can be done under control. They demonstrated greater activity than the free enzyme, and are more stable than the free enzyme [13].

Organic matter is typically classified as either a growth substrate or a non-growth substrate due to the complexity of the wastewater's components. The growth substrate acts as an energy and carbon source, supporting cell growth and increasing the non-growth substrate’s transformation rate. However, it is essential to investigate the ability of enzymes and microbial cultures to biotransform CPs in wastewater under these conditions.

Several *Bacillus* specie*s* are well known for the biodegradation of different phenolic compounds [14, 15]. *Bacillus subtilis* is an aerobic, endospore-forming, Gram-positive bacteria, opportunistic pathogen, and the virulence characteristics of the microorganism are low [16]. Several authors have investigated *Bacillus subtilis* cells ability to degrade the 2,4-DCP [17–19]

Therefore, the current work aimed at biotransformation of 2,4-DCP using immobilized safe *Bacillus subtilis* AAK cultures and immobilized its laccase enzyme. Moreover, wastewater management applying both immobilized bacterial cultures, and laccase enzymes will be investigated.

## Methods

### Microbial strain

*Bacillus subtilis* AAK strain, which was previously isolated, identified as a marine halophilic 2,4-DCP-degrading bacterial strain and kept in GenBank (accession No. MF 037698), was used in this study [19].

### Assessment of hemolytic activity

The hemolytic activity of *B. subtilis* AAK strain was detected according to the method described by Brutscher et al. [20]. The cells were streaked onto blood agar plates to test their ability to lyse blood cells. The plates were incubated at 30 °C for 24 h. After incubation, the blood agar was inspected for an alpha, beta, or gamma-hemolysis. Alpha-hemolysis, or incomplete hemolysis, is indicated by a discolored, darkened, or green medium color after testing culture growth. Complete hemolysis, or beta-hemolysis, is referred to as a clear and colorless medium after growth. An indiscernible change in the color of the agar indicates that no hemolysis occurred (i.e., gamma-hemolysis).

### Growth and culturing conditions

The bacterial isolate *Bacillus subtilis* AAK strain could use 2,4-DCP (300 mg/l) as the sole carbon and energy source. The minimal mineral synthetic (MMS) medium [19] was sterilized at 121 °C for 15–20 min by autoclaving, and sterilized 2,4-DCP (300 mg/l) was added to the medium after sterilization. Growth was carried out in 250 ml Erlenmeyer flasks containing 50 ml culture medium at 37 °C on an orbital shaker (160 rpm) for 72 h. Growth of bacteria was detected as optical density (OD) by spectrophotometer (600 nm). The resulted culture was used in subsequent experiments.

### Determination of residual 2,4-DCP

Residual 2,4-DCP was measured through spectrophotometry using 4-aminoantipyrine solution and ferricyanide solution according to the method mentioned by Yang and Humphery [21] and Huang et al. [22]. The resulting color was measured at 510 nm, and the concentration of residual 2,4-DCP was calculated from the standard curve of different 2,4-DCP concentrations ranging from 10 to 100 mg/ml.

### Laccase activity assay

Using ABTS (2, 2′-azino-bis (3-ethylbenzthiazoline-6-sulphonic acid) as a substrate, laccase activity assay was determined [23]. The mixture consisted of the enzyme solution, ABTS (0.01 M), and 50 mM phosphate buffer pH 6.5. The mixture was incubated for 15 min at 35 °C. After incubation, trichloroacetic acid (w/v) was added to stop the reaction. The laccase activity was calorimetrically estimated at 420 nm. Enzyme activity is the amount of enzyme that oxidizes one mol of substrate per ml (one unit).

### Immobilization of *B. subtilis* AAK cultures

#### By entrapment

Liquid cultures (50 ml) were centrifuged for 30 min at 6000 rpm, and the clear supernatant was discarded. The cell pellets were suspended in a sterilized solution of Na alginate (3%) or *k*-carrageenan (3%). The mixture was added dropwise with a sterile syringe (20 ml) fitted with a wide bore needle (one mm diameter) into a sterilized 2% CaCl_2_ (for alginate) and 2% KCl (for *k*-carrageenan), and beads were formed immediately [24]. The beads were left for 2 h to harden before being filtered and harvested. The formed beads were collected and washed with 50 mM phosphate buffer (pH 7.5) to remove the excess Ca^2+^ or K^+^. The gel materials, including agarose and agar, were also used for the entrapment of cells. Each material (3% (wt/vol)) was mixed with cell suspension after cooling to about 45 °C. The mixture was poured into sterilized Petri dishes, allowed to solidify and cut into uniform-sized particles. Each biocatalyst was added to 50 ml of fresh, sterilized medium and incubated under shaking conditions at 37 °C. One ml of entrapped and free bacterial culture was withdrawn at different incubation times and analyzed for residual 2,4-DCP concentration.

#### Adsorption to solid supports

As solid supports for the adsorption of cells, polyurethane foam, stone particles, ceramic cubes, luffa pulp, nut particles, and charcoal cubes were used. Each solid support was added to 50 ml of *B. subtilis* AAK culture (OD_600_= 1.0) [25]. Afterward, the cultures were left for 4 h under shaking (80 rpm) at room temperature to allow good adsorption of bacterial cells to the carrier material (wet formulation). The support materials were removed from the flask and washed with sterile double distilled water to remove any cells that were not sufficiently adherent to the carrier material. Under optimal conditions, the sterilized medium and support materials were incubated. At different incubation periods, 1 ml of free and adsorbed culture was withdrawn and analyzed for residual 2,4-DCP concentration.

#### Reuse of immobilized *B. subtilis* AAK cells

The medium containing immobilized culture onto suitable material support was incubated at 37 °C. The immobilized cells were reused by removing the culture medium, a fresh, sterilized medium was added, and a new cycle was run. The process was repeated several times. At the end of each cycle, the rest of the 2,4-DCP concentration was estimated.

#### Scanning electron microscopy (SEM)

Scanning electron micrograph of the immobilized *B. subtilis* AAK cells was performed using SEM (JEOL JEM- 2100 F (JEOL Ltd, Japan).

#### Partial purification of laccase enzyme

Ammonium sulfate was added to the culture filtrate to obtain various fractions (35, 50, 65, 75, 85, and 95% saturation). Each precipitate was dissolved in 50 mM phosphate buffer (pH 7.5) and dialyzed against distilled water in a refrigerator overnight [26]. After dialysis, each fraction’s enzyme activity and protein content were determined.

### Immobilization of laccase enzyme

#### Physical adsorption

Different carriers, such as silica gel and charcoal, were used for the adsorption of laccase enzymes. The carrier was incubated with the partially purified enzyme (159.4 U *B. subtilis* AAK laccase) dissolved in 50 mM phosphate buffer (pH 7.5) at 4 °C overnight. The enzyme activity and protein of unbound and immobilized enzymes were determined [27].

#### Ionic binding

Anion exchanger (DEAE-cellulose) equilibrated with phosphate buffer (0.05 mol/1, pH 7.5) was incubated with the partially purified enzyme (159.4 U *B. subtilis* AAK laccase) dissolved in the same buffer for 12 h at 4 °C. The protein content and enzyme activity of bound (immobilized) and unbound were estimated [27].

#### Entrapment

The partially purified enzyme (159.4.2 U of *B. subtilis* AAK laccase) was mixed with 5ml of Na-alginate of different concentrations (2, 3, and 5% (w/v)). The entrapment process was conducted by pouring the mixture into a sterilized 25 ml mol/1 CaCl_2_ solution. The resulting beads (1.5–2.0 mm diameter) were collected and washed with distilled water to remove the unbound enzyme [27].

### Analysis of industrial effluent

The wastewater sample (effluent) was collected from the Rikta-Paper industry, Alexandria, Egypt. Different physicochemical properties of the collected effluent were analyzed (Table 1). Before and after degradation, the temperature, pH, chemical oxygen demand (COD), biological oxygen demand (BOD), and total organic compounds (TOC) analysis of the sample were performed [28].

**Table 1:** Temperature, pH, BOD, COD, TOC, and total phenol in wastewater sample

Parameter	
**pH**	7.57
**temperature (°C)**	28.10
**BOD (**mg O_2_/l)	398
**COD (**mg O_2_/l)	1290
**TOC (**mg C/l)	460
**Total phenol** (mg/l)	50

### 2,4-DCP biotransformation experiment

The wastewater sample was sterilized by autoclaving for 15–20 min at 121 °C and left to cool. About 300 ml of sterilized wastewater sample and 60 pieces of luffa pulp-containing cells were placed in the air-bubble column. Subsequently, a second column was inoculated with immobilized *B. subtilis* AAK laccase. The wastewater samples were incubated under optimal conditions. Before and after incubation, a predetermined volume of samples was aseptically drawn at time intervals and analyzed for residual 2,4-DCP, TOC, BOD, and COD. Samples without the immobilized bacterial strain or enzyme were considered a control [23].

$$\mathrm{Removal}\ \mathrm{efficiency}\ \left(\%\right)\ \left(\mathrm{BOD}\right)=\left(\mathrm{initial}\ \left(\mathrm{BOD}\right)\ \mathrm{of}\ \mathrm{the}\ \mathrm{wastewater}-\mathrm{final}\ \left(\mathrm{BOD}\right)\ \mathrm{after}\ \mathrm{removal}/\mathrm{initial}\ \left(\mathrm{BOD}\right)\ \mathrm{of}\ \mathrm{the}\ \mathrm{wastewater}\right)\times 100$$

$$\mathrm{Removal}\ \mathrm{efficiency}\%\,\left(\mathrm{COD}\right)=(\left(\mathrm{initial}\ \left(\mathrm{COD}\right)\ \mathrm{of}\ \mathrm{the}\ \mathrm{wastewater}-\mathrm{final}\ \left(\mathrm{COD}\right)\ \mathrm{after}\ \mathrm{removal}/\mathrm{initial}\ \left(\mathrm{COD}\right)\ \mathrm{of}\ \mathrm{the}\ \mathrm{wastewater}\right)\times 100$$

$$\mathrm{Removal}\ \mathrm{efficiency}\%\,\left(\mathrm{TOC}\right)=\left(\mathrm{initial}\ \mathrm{TOC}\right)\ \mathrm{of}\ \mathrm{the}\ \mathrm{wastewater}-\mathrm{final}\ \left(\mathrm{TOC}\right)\ \mathrm{after}\ \mathrm{removal}/\mathrm{initial}\ \left(\mathrm{TOC}\right)\ \mathrm{of}\ \mathrm{the}\ \mathrm{wastewater})\times 100$$

### Statistical analysis

All experiments were performed in triplicate, and data were recorded as mean values ± standard deviation (SD).

## Results

### Blood hemolysis of *B. subtilis* AAK cells

*B. subtilis* AAK cells were streaked onto blood agar plates to characterize any potential hemolytic activity. The strain was γ-hemolytic (no clear halo around the bacterial colonies, Supplementary Fig. 1). The γ-hemolytic *Bacillus subtilis* AAK strain was selected as non-pathogenic.

### Biotransformation of 2,4-DCP by cell immobilization

**Fig. 1:**
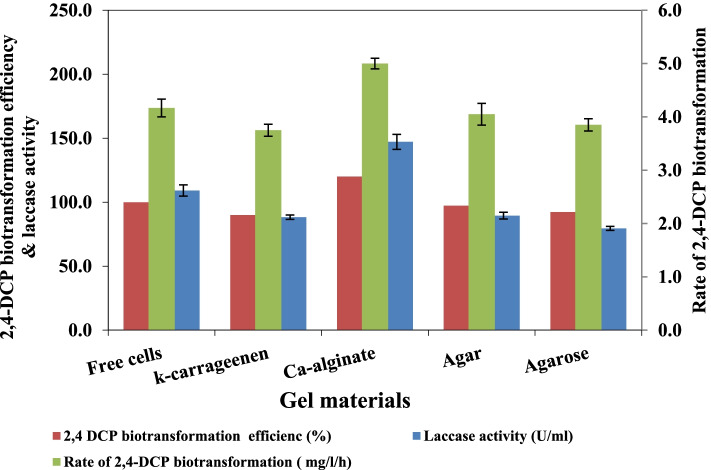
Biotransformation of 2,4-DCP by *B. subtilis* AAK entrapped cells of with different gel materials. Data are represented as mean ±SD across technical replicates (*n* = 3)

**Fig. 2:**
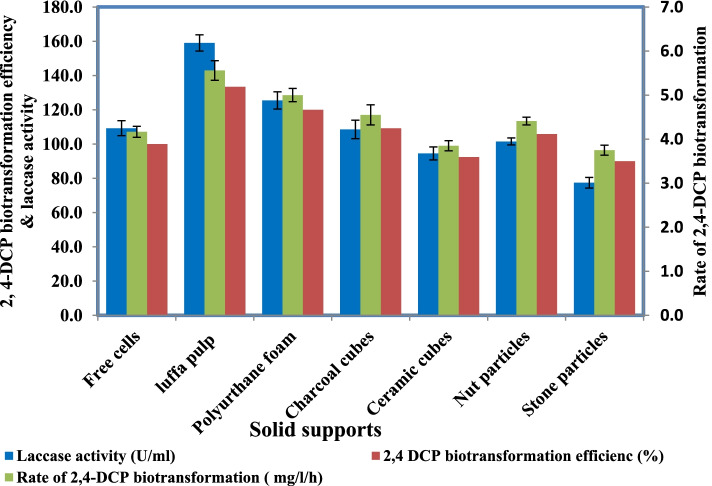
Biotransformation of 2,4-DCP by *B. subtilis* AAK cells adsorbed with different solid supports. Data are represented as mean ±SD across technical replicates (*n* = 3)

2,4-DCP biotransformation using different gel materials was investigated as graphically in Fig. 1. The biocatalyst formed by entrapment in alginate beads gave the maximum 2,4-DCP biotransformation rate (5.0 mg /l/h) and laccase activity (147.2 U/ml) which recorded 1.20-fold and 1.35-fold, respectively, increase compared to free cells. Immobilization by adsorption using different support materials (Fig. 2) was also studied. It is evident that luffa pulp and polyurethane foam revealed the maximum 2,4-DCP biotransformation rate (5.56 mg/l/h and 5.0 mg/l/h, respectively) and laccase activity (159.05 U/ml and 125.45 U/ml, respectively). Adsorbed cells on luffa pulp recorded higher 2,4-DCP biotransformation compared to free and entrapped cells, and the results are significant (*P* < 0.05). In addition, no decrease in 2,4-DCP concentration was detected when using support material without viable cells (biocatalysts) (data not shown), indicating that 2,4-DCP removal was due to biotransformation by bacterial cells and not due to adsorption or entrapment.

### Biotransformation of different phenolic compounds (PCs) by free and immobilized *B. subtilis* AAK cells

**Fig. 3:**
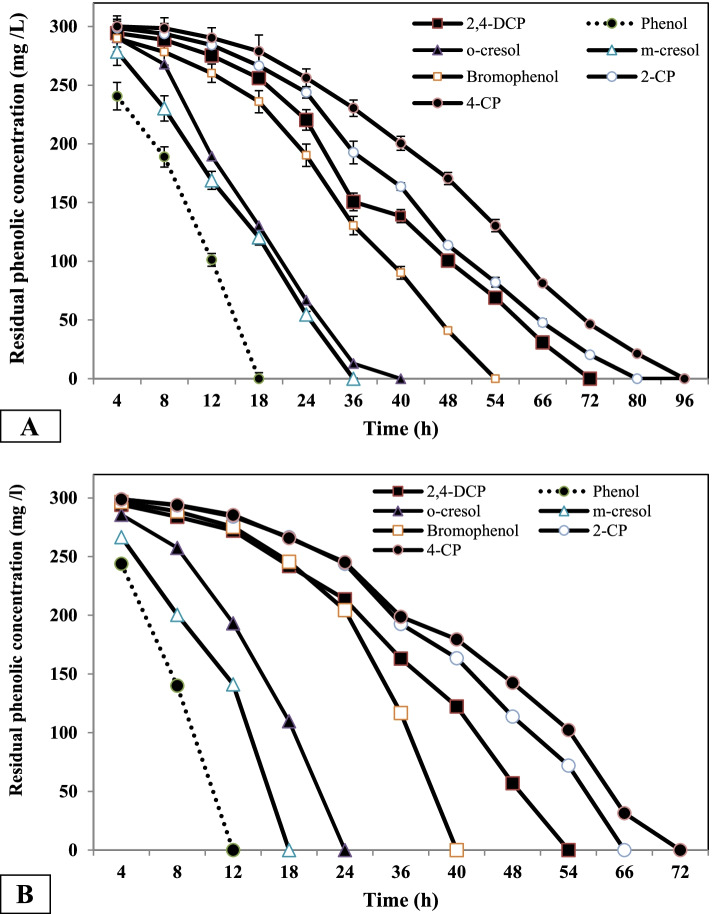
Biotransformation of different PCs by free (**A**) and immobilized cultures (**B**) at different time interval. Data are represented as mean ±SD across technical replicates (*n* = 3)

Phenol, o-cresol, m-cresol, bromophenol, 2,4-DCP, and 2-CP were used as examples of PCs (300 mg/l) to model the PC treatment of wastewater. The results (Fig. 3B) revealed that the immobilized *B. subtilis* AAK culture could simultaneously degrade all the added PCs. Phenol, m-cresol, and o-cresol were depleted within 12 h, 18 h, and 22 h, respectively. In contrast, bromophenol, 2,4-DCP, 2-CP, and 4-CP were significantly prolonged (40 h, 54 h, 66 h, and 72 h, respectively). The maximum degradation by free cells (Fig. 3A) of phenol, o-cresol, m-cresol, bromophenol, 2,4-DCP, 2-CP, and 4-CP was detected at 18 h, 38 h, 36 h, 54 h, 80 h, and 96 h, respectively, which was higher than immobilized cultures and the results are significant (*P* < 0.05).

### Partial purification and laccase immobilization

The partially purified *B. subtilis* AAK laccase enzyme was partially purified by fractional precipitation with ammonium sulfate (85%), yielding the highest laccase activity at about 3.01-fold purification. Additionally, immobilization of *B. subtilis* AAK laccase was conducted on different carriers, and its activity was estimated (Table 2). Enzyme immobilized in 3.0% alginate beads exhibited the highest activity (356.70 U/g of carriers) and the highest immobilization yield (93.64%). Therefore, alginate beads (3%) were determined to be the optimal carrier for future research.

**Table 2:** Immobilization of partially purified *B. subtilis* AAK laccase enzyme on different materials

Carrier	Added enzyme (A)	Unbound enzyme (B)	Bound enzyme (I)	Specific activity of the immobilized enzyme (U/mg protein)	Immobilization yield I/(A-B)%
**Ionic binding**					
Silica gel	478.2	162.66	236.7	75.14	75.01
Charcoal cubes	478.2	132.06	235.98	74.91	68.17
**Ionic binding**					
DEAE-cellulose	478.2	69.30	268.17	85.13	65.58
**Entrapment**					
Alginate 2%	478.2	90.66	319.5	101.43	82.44
Alginate 3%	478.2	97.29	356.7	113.24	93.64
Alginate 5%	478.2	115.95	331.05	105.10	91.39

### Reuse of adsorbed *B. subtilis* AAK cells and laccase enzyme

**Fig. 4:**
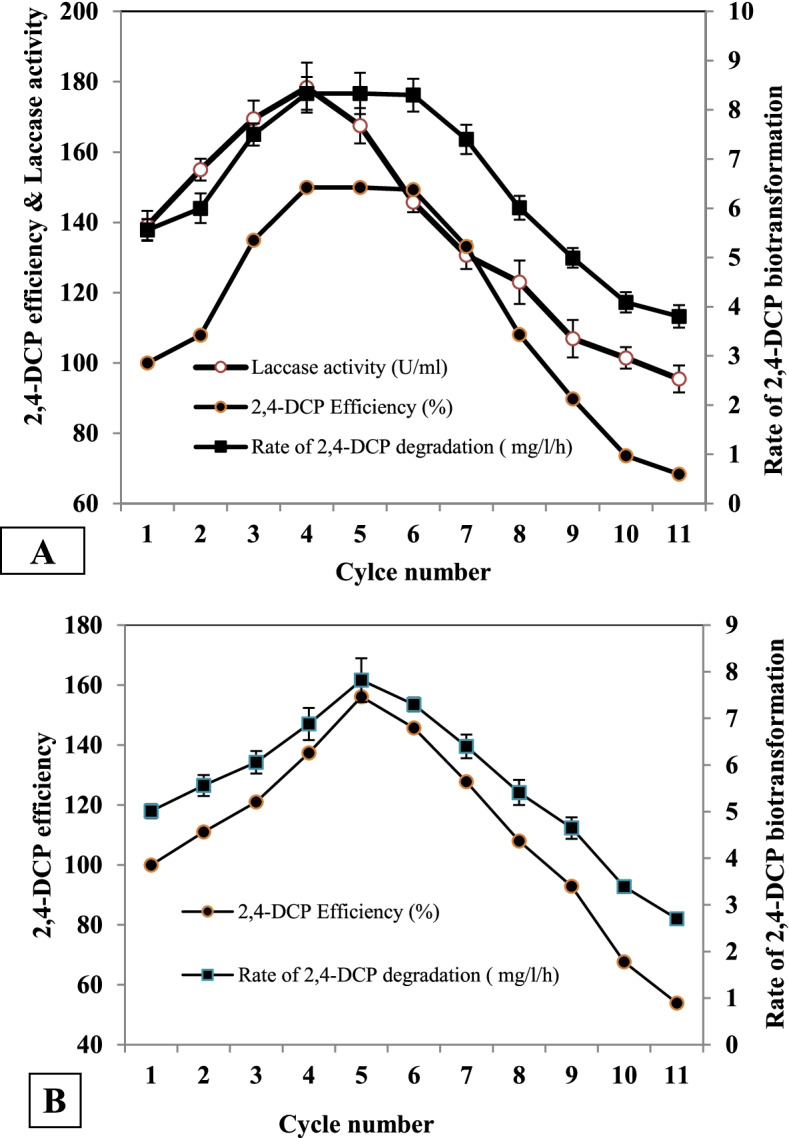
Biotransformation of 2,4-DCP by the reused adsorbed *B. subtilis* AAK cultures (**A**) and immobilized laccase enzyme (**B**). Data are represented as mean ±SD across technical replicates (*n* = 3)

The data (Fig. 4A, B) revealed that the biotransformation rate gradually increased by reusing immobilized cultures and enzymes, reaching a maximum value in the 6th and 4th reuse (for cells and enzyme, respectively), which were 1.49-fold and 1.59 -fold of the first run.

Removal of 2,4-DCP by immobilized laccase led to 100% removal of 2,4-DCP after 40 h of incubation. On the contrary, free laccase led to 100% 2,4-DCP removal after 48 h (data is not shown). By reusing immobilized cells and enzymes, the biotransformation rate decreased to 3.8 mg/l/h and 4.7 mg/l/h at the 11th cycle, respectively, with significant differences (*P* < 0.05).

**Fig. 5:**
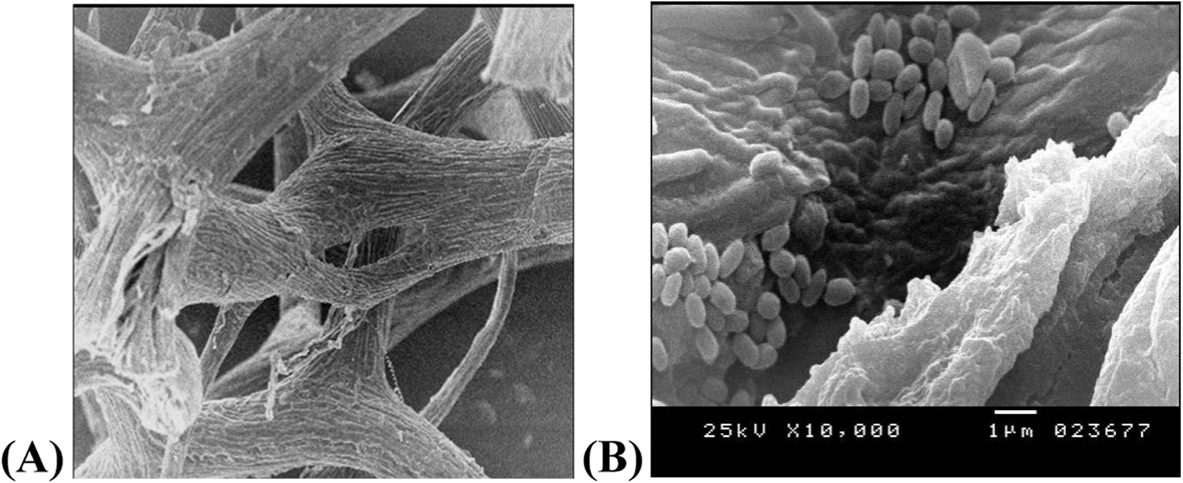
Electron microscope micrographs showing the structure of luffa pulp (**A**). The density of cells of *B. subtilis* AAK adsorbed on the surface of luffa pulp surface (**B**)

The supporting material with attached bacterial cells was observed in the SEM. SEM analysis confirmed the successful cells of *B. subtilis* AAK to luffa pulp (Fig. 5), indicating the good adsorption of cells to the surface of supporting material.

### The efficiency of immobilized *B. subtilis* AAK cultures and laccase enzyme for wastewater treatment

The reduction of contaminants in wastewater by using immobilized *B. subtilis* AAK culture and its laccase were estimated. Furthermore, BOD, COD, and TOC were detected before and after wastewater treatment. The maximum reduction efficiencies (Table 3) of BOD, COD, TOC, and 2,4-DCP were 92.05%, 90.11%, 75.88%, and 81.05%, respectively, after 52 h when using immobilized cells. However, the highest reduction efficiencies of BOD, COD, TOC, and 2,4-DCP were 90.12%, 82.66%, 68.9%, and 64.34%, respectively, after 56 h of incubation using free cells.

**Table 3:** Removal of some pollutants and 2,4-DCP by free and immobilized *B. subtilis* AAK cells from wastewater sample. Data are expressed as mean ± standard deviation across technical replicates (*n* = 3)

Incubation time (h)	Immobilized cells				Free cells			
	Removal efficiency (%)				Removal efficiency (%)			
	2,4-DCP	BOD	COD	TOC	2,4-DCP	BOD	COD	TOC
**12**	21.85±0.05	12.50±0.06	17.90±0.05	18.5±0.05	12.15±0.06	14.10±0.07	8.11±0.06	10.5±0.17
**24**	40,39±0.04	28.30±0.08	34.40±0.15	34.6±0.12	26.02±0.04	26.30±0.09	20.56±0.10	19.00±0.10
**36**	60.34±0.04	49.66±0.09	50.50±0.11	55.7±0.08	52.30±0.06	35.66±0.08	30.00±0.12	29.99±0.11
**40**	75.67±0.32	71.45±0.10	56.50±0.10	70.3±0.10	68.99±0.13	48.45±0.10	40.17±0.04	42.18±0.07
**44**	80.44±0.16	87.52±0.06	64.69±0.03	75.11±0.12	76.90±0.09	65.11±0.05	52.09±0.20	50.55±0.13
**48** **52** **56**	89.07±0.04 90.05±0.06 90.05±0.09	90.11±0.06 90.11±0.04	71.09±0.08 75.88±0.06 75.88±0.10	79.55±0.12 81.05±0.07 81.06±0.09	80.01±0.11 85.12±0.6 90.12±0.05	72.00±0.11 77.66±0.06 82.66±0.07	60.56±0.13 67.90±0.08 68.90±0.21	60.50±0.24 63.34±0.19 64.34±0.10

The maximum reduction efficiencies of BOD, COD, TOC, and 2,4-DCP were 95%, 93%, 90.11%, and 85.55%, respectively, after 48 h of incubation using an immobilized enzyme (Table 4). Moreover, the highest reduction efficiencies of BOD, COD, TOC, and 2,4-DCP were 92.11%, 82.0%, 88.67%, and 74.8%, respectively, after 54 h of incubation using a free enzyme.

**Table 4:** Removal of some pollutants and 2,4-DCP by free, immobilized laccase from wastewater sample. Data are expressed as mean ± standard deviation across technical replicates (*n* = 3)

Incubation time (h)	Immobilized laccase enzyme				Free laccase enzyme			
	Removal efficiency (%)				Removal efficiency (%)			
	2,4-DCP	BOD	COD	TOC	2,4-DCP	BOD	COD	TOC
**12**	25.94±0.08	14.34±0.09	18.90±0.26	20.0±0.12	10.64±0.18	18.08±0.26	10.56±0.29	16.0±0.18
**24**	45.05±0.08	32.50±0.10	37.5±0.17	36.5±0.19	20.01±0.17	29.11±0.18	23.90±0.26	26.5±0.22
**36**	65.50±0.10	51.44±0.11	53.60±0.21	59.0±0.12	45.0±0.38	39.14±0.19	32.60±0.24	37.01±0.23
**40**	72.12±0.11	68.30±0.23	60.34±0.19	60.2±0.22	65.66±0.22	54.00±0.22	45.34±0.13	45.26±0.19
**44**	89.55±0.12	76.12±0.11	69.20±0.10	65.5±0.19	72.55±0.18	65.10±0.13	52.17±0.11	54.01±0.21
**48**	92.01±0.06	89.50±0.17	84.19±0.08	75.6±0.19	80.11±0.14	72.45±0.52	63.10±0.07	65.44±0.27
**50** **54**	95.01±0.06 95.00±0.11	90.0±0.14 93.0±0.08	90.11±0.09 90.11±0.08	85.6±0.33 85.6±0.49	86.11±0.11 92.11±0.17	79.0±0.21 82.0±0.08	72.11±0.19 88.67±0.21	70.50±0.10 74.80±0.35

## Discussion

Classical methods for removing phenolic compounds have many disadvantages; therefore, biological methods are considered a good alternative. Bioremediation is a significant ecologic technology that uses microorganisms or microbial enzymes to degrade many environmental pollutants efficiently. The present study was carried out to optimize the 2,4-DCP biotransformation process by immobilizing cells and laccase enzymes, along with modeling bioremediation techniques for wastewaters.

In the biotransformation of 2,4-DCP, as an example of chlorophenols, various promising approaches are presented. Most magnitude utilize immobilized cells (*B. subtilis* AAK) and its laccase enzyme, with a higher rapid rate of 2,4-DCP biotransformation efficiency and performance of the process under aerobic conditions.

Few reports have thoroughly examined the biotransformation of 2,4-DCP by immobilized bacterial laccase. Entrapment and adsorption techniques were applied to approach the biotransformation of 2,4-DCP by immobilized *B. subtilis* AAK cells, and due to the increased availability of 2,4-DCP (substrate) to microbial cells, the results revealed that adsorption of cells detected a high biotransformation rate. On the contrary, the cells diffused in the Ca-alginate matrix led to slow diffusion of the substrate (2,4-DCP) and air into the polymer beads, which may decrease the 2,4-DCP biotransformation rate. Another study found that the efficiency of an immobilization process may depend on the support used [29]. Many authors reported enhanced biotransformation of different phenolic and aromatic compounds applying immobilized microbial cells [30–32].

The efficiency of immobilized *B. subtilis* AAK cultures in degrading different phenolic compounds was investigated. The results indicated that the biotransformation of phenol, o-cresol, m-cresol, bromophenol, 2,4-DCP, 2CP, and 4CP by immobilized cultures was higher than in free cultures. Our findings are in agreement with Yordanova et al. [33], who found that the degradation of phenol and 2,4-DCP by immobilized cultures was faster than 2-CP and 4-CP degradation. In addition, numerous reports demonstrated that the immobilized cultures process the highest removal potency for different CPs than free cultures [34–36].

Microbial laccases immobilization has recently increased the viability for their industrial uses such as reuse, easy recovery, and enhanced stability. Different carriers or supports for the immobilization of laccase have been reported [37, 38]. Several investigations covered the laccases produced by fungi (production, immobilization, and application) rather than bacterial laccases, which may be contributed to the highest potential for oxireduction of fungal laccases [9] compared to bacterial laccases. Therefore, this report may provide more information about immobilized bacterial laccase derived from marine halophilic *B. subtilis* AAK for efficient biotransformation of some phenolic compounds in a contaminated environment. Ammonium sulfate (85%) as a precipitant [39] and 3% alginate (356.70 U/g carrier) as an immobilization carrier were found to be the most favorable conditions for immobilizing *B. subtilis* AAK laccase. Due to its low cost and biodegradability, alginate was utilized as a common carrier for immobilizing many enzymes [40, 41].

Multiple batch fermentations were conducted to investigate the long-term stability of the catabolic process of 2,4-DCP biotransformation by immobilized cells and enzymes. The high efficiency of the immobilized enzyme, which may be attributable to Bacillus-produced laccases, is a component of their endospore coat, which protects cells from external stress and high concentrations of toxic substances [42]. The results of the present investigation also showed that the immobilized cultures and enzymes were relatively stable for a long time. The reused immobilized cultures and enzymes can be considered a crucial parameter in an industrial treatment, which determines the effectiveness of biotransformation over time [36]. Luffa pulp (as an adsorbed solid support) recorded lower cell leakage, maximum mechanical stability, and resistance to biological and chemical degradation, making it suitable for an extended period of repetition [43]. The obtained results partially agree with Bagewadi et al. [40] and Wen et al. [44].

In addition, the current work attempted to present a different perspective on the bioremediation of the phenolic contaminated environmental sites. The novelty of this method is confined to using immobilized cells and enzymes to direct bioremediation of 2,4-DCP under aerobic conditions at room temperature.

The production process of paper and pulp is a water-intensive process that generates a large amount of wastewater characterized by a high concentration of suspended solids (SS), COD, TOC, and BOD. The reduction efficiency of the examined parameters at room temperature may be considered economical, representing a low-energy biological technique compared to the classical techniques that need high energy and temperature levels.

The data revealed that the immobilized enzyme demonstrated an increased removal rate of 2,4-DCP than that of immobilized cells. Subsequently, immobilized enzyme techniques have more benefits than all microbial cell methods due to some enzyme characteristics such as easier handling, highest specificity, and its activity can be better standardized/optimized based on the environment [45]. Additionally, numerous studies focused on the increase of phenol biotransformation and its chlorinated derivatives by immobilized cells [46].

The higher biodegradation efficiency of 2,4-DCP in wastewater samples (Tables 3 and 4) was detected using immobilized enzymes than that of free enzymes and cells. These results are in line with Zhang et al. [47] and Chen et al. [48], who applied laccases immobilized in nanomaterials for the biodegradation of chlorophenol. Another study found that laccases (free or immobilized) demonstrated high biotransformation of 2,4-dinitrophenol in soil [8]. In the environment (soil and water), many studies used laccases for enzymatic biodegradation of different organic pollutants [49]. In addition, biodegradation of different PCs, pesticides, drugs, organic pollutants [50], synthetic dyes, and others in water and soil by immobilized and free laccases was investigated [44].

## Conclusion

The present data support the potential of using immobilized safe marine halophilic *B. subtilis* AAK strain and its laccase enzyme as potential approaches for the 2,4-DCP removal. This method is a cost-effective alternative for large-scale wastewater treatment. Future prospective work would focus on determining the viability of applying the immobilized enzyme approach to biodegrade different phenolic compounds on a larger scale in different environments. Furthermore, cloning and over-expression of the gene responsible for 2,4-DCP biotransformation will also be addressed.

## Supplementary Information

**Additional file 1: Supplementary Figure 1.** Gamma hemolysis of *Bacillus subtilis* AAK (did not shown any hemolytic activity) against blood in the blood agar medium.

## Data Availability

All data generated or analyzed during this study are included in this published article.

## Abbreviations

2,4-DCP: 2,4-Dichlorophenol
BOD: Biological oxygen demand
COD: Chemical oxygen demand
MMS: Minimal mineral synthetic
mg/l/h: Milligram per liter per hour
OD: Optical density
ppm: Part per million
TOC: Total organic compounds
rpm: Round per minute
U/g: Unit per gram
wt/vol: Weight per volume

## References

[1] Tobajas M, Monsalvo VM, Mohedano AF (2012). Enhancement of cometabolic biodegradation of 4-chlorophenol induced with phenol and glucose as carbon sources by *Comamonas testosterone*. Environ Manag.

[2] Li JW, Cai WJ, Zhu L (2011). The characteristics and enzyme activities of 4-chlorophenol biodegradation by *Fusarium* sp. Bioresour Technol.

[3] Gupta P, Sreekrishnan TR, Shaikh ZA (2018). Evaluating the effects on performance and biomass of hybrid anaerobic reactor while treating effluents having glucose with increasing concentrations of 4-chlorophenols. Environ Chem Eng.

[4] Ra JS, Oh SY, Lee BC, Kim SD (2008). The effect of suspended particles coated by humic acid on the toxicity of pharmaceuticals, estrogens and phenolic compounds. Environ Int.

[5] Geed SR, Kureel MK, Giri BS, Singh RS, Rai BN (2017). Performance evaluation of Malathion biodegradation in batch and continuous packed bed bioreactor (PBBR). Bioresour Technol.

[6] Barzkar N (2020). Marine microbial alkaline protease: an efficient and essential tool for various industrial applications. Int J Biol Macromol.

[7] Kurniawati S, Nicell JA (2008). Characterization of *Trametes versicolor* laccase for the transformation of aqueous phenol. Bioresour Technol.

[8] Georgieva S, Godjevargova T, Mita DG (2010). Non-isothermal bioremediation of waters polluted by phenol and some of its derivatives by laccase covalently immobilized on polypropylene membranes. J Mol Catal B Enzym.

[9] Rahmani H, Lakzian A, Karimi A, Halajnia A (2020). Efficient removal of 2,4-dinitrophenol from synthetic wastewater and contaminated soil samples using free and immobilized laccases. J Environ Manag.

[10] Mot AC, Coman C, Hadade N (2020). Yellow laccase from *Sclerotinia sclerotiorum* is a blue laccase that enhances its substrate affinity by forming a reversible tyrosyl-product adduct. PLoS One.

[11] Janusz G, Pawlik A, Świderska-Burek U et al (2020) Laccase properties, physiological functions, and evolution. Int J Mol Sci 21(3):966 10.3390/ijms21030966 PMC7036934 32024019

[12] El-Naas MH, Al-Zuhair S, Alhaija MA (2010). Removal of phenol from petroleum refinery wastewater through adsorption on date-pit activated carbon. Chem Eng J.

[13] Nadaroglu H, Sonmez Z (2016). Purification of an endo-beta 1,4-mannanase from *Clitocybe geotropa* and immobilization on chitosan-coated magnetite nanoparticles: Application for fruit juices. Digest J Nano Biostruct.

[14] Tallur P, Megadi V, Kamanavalli C, Ninnekar H (2006). Biodegradation of p-cresol by *Bacillus* sp. strain PHN 1. Curr Microbiol.

[15] Hasan SA, Jabeen S (2015). Degradation kinetics and pathway of phenol by *Pseudomonas* and *Bacillus* species. Biotechnol Biotechnol Equip.

[16] Boer AS, Diderichsen B (1991). On the safety of *Bacillus subtilis* and *B. amyloliquefaciens*: a review. Appl Microbiol Biotechnol.

[17] Hassan H, Schulte-Illingheim L, Jin B, Dai S (2016). Degradation of 2,4-dichlorophenol by *Bacillus Subtilis* with concurrent electricity generation in microbial fuel cell. Procedia Eng.

[18] Sandhibigraha S, Chakraborty S, Bandyopadhyay T, Bhunia B (2020). A kinetic study of 4-chlorophenol biodegradation by the novel isolated *Bacillus subtilis* in batch shake flask. Environ Eng Res.

[19] Farag AM, Fawzy A, El-Naggar MY, Ghanem KM (2021). Biodegradation and enhancement of 2,4-dichlorophenol by marine halophilic *Bacillus subtilis* AAK. Egy J Aquatic Res.

[20] Brutscher LM, Borgmeier C, Garvey SM, Spears JL (2022) Preclinical safety assessment of *Bacillus subtilis* BS50 for probiotic and food applications. Microorganisms 10:1038 10.3390/microorganisms10051038 PMC9144164 35630480

[21] Yang RD, Humphery AE (1975). Dynamic and steady sate studies of phenol biodegradation in pure and mixed cultures. Biotechnol Bioeng.

[22] Huang Y, Xi Y, Yang Y, Chen C (2014). Degradation of 2,4-dichlorophenol catalyzed by the immobilized laccase with the carrier of Fe3O4@MSS–NH2. Chin Sci Bull.

[23] Sarnthima R, Khammuang S (2007). Laccase from spent mushroom compost of *Lentinus polychrous* Lev. and its potential for remazol brilliant blue R decolourisation. Biotechnol.

[24] Mohanty SS, Jena HM (2017). Biodegradation of phenol by free and immobilized cells of a novel *Pseudomonas* sp. NBM11. Braz J Chem Eng.

[25] Farag AM, Hassan SW, Beltagy EA, El-Shenawy MA (2015). Optimization of production of anti-tumor L-asparaginase by free and immobilized marine *Aspergillus terreus*. Egy J Aquatic Res.

[26] Javed MR, Rashid MH, Nadeem HU, Riaz M, Perveen R (2009). Catalytic and thermodynamic characterization of endoglucanase (CMCase) from *Aspergillus oryzae* cmc-1. Appl Biochem Biotechnol.

[27] Farag AM, Hassan MA (2004). Purification, characterization and immobilization of a keratinase from *Aspergillus oryzae*. Enzym Microb Technol.

[28] APHA (American Public Health Association) (1992) Standard Methods for the Examination of Water and Wastewater. (18th ed.), Washington, DC

[29] Musa N, Latip W, Abd Rahman RNZ, Salleh A, Ali MSM (2018) Immobilization of an antarctic *Pseudomonas* AMS8 lipase for low temperature ethyl hexanoate synthesis. Catalysts 8(6):234

[30] Tosu P, Luepromchai E, Suttinun O (2015). Activation and immobilization of phenol degrading bacteria on oil palm residues for enhancing phenols degradation in treated palm oil mill effluent. Environ Eng Res.

[31] Gujjar P, Jain L (2018). Isolation of trinitrophenol (TNP) degraders and its application in bioremediation. Int J Pharm Bio Sci.

[32] Farsi RM, Alaidaroos BA, Alharbi NM, Basingab FS, Nass NM, Hassoubah SA (2021). Biodegradation of picric acid (2,4,6-trinitrophenol, TNP) by free and immobilized marine *Enterococcus thailandicus* isolated from the Red Sea, Saudi Arabia. Egy J Aquatic Res.

[33] Yordanova G, Godjevargova T, Nenkova R, Ivanova D (2013). Biodegradation of phenol and phenolic derivatives by a mixture of immobilized cells of *Aspergillus Awamori* and *Trichosporon Cutaneum*. Biotechnol Biotechnol Equip.

[34] Jiang B, Zhou Z, Dong Y (2015). Bioremediation of petrochemical wastewater containing BTEX compounds by a new immobilized bacterium *Comamonas* sp. JB in magnetic gellan gum. Appl Biochem Biotechnol.

[35] Hou J, Liu F, Wu N, Ju J, Yu B (2016). Efficient biodegradation of chlorophenols in aqueous phase by magnetically immobilized aniline degrading *Rhodococcus rhodochrous* strain. J Nanobiotechnol.

[36] Lin YH, Cheng YS (2020). Phenol degradation kinetics by free and immobilized *Pseudomonas putida* BCRC 14365 in batch and continuous-flow bioreactors. Processes.

[37] Daronch NA, Pereira CS (2020). Elucidating the choice fora precise matrix for laccase immobilization: a review. Chem Eng J.

[38] Alvarado-Ramirez L, Kelbert M, Rostro-Alanis J, Rodriguez-Rodriguez (2021). Exploring current tendencies in techniques and materials for immobilization of laccases- a review. Int J Biol Macromol.

[39] Zerva A, Pentari C, Topakas E (2020). Crosslinked enzyme aggregates (CLEAs) of laccases from *Pleurotus citrinopileatus* induced in olive oil mill wastewater (OOMW). Molecules.

[40] Bagewadi ZK, Mulla SI, Ninnekar HZ (2017). Purification and immobilization of laccase from *Trichoderma harzianum* strain HZN10 and its application in dye decolorization. J Genet Eng Biotechnol.

[41] Olajuyigbe FM, Adetuyi OY, Fatokun CO (2019). Characterization of free and immobilized laccase from *Cyberlindnera fabianii* and application in degradation of bisphenol A. Int J Biol Macromol.

[42] Wu J, Yu HQ (2007). Biosorption of 2,4-dichlorophenol by immobilized white rot fungus *Phanerochaete chrysosporium* from aqueous solutions. Bioresour Technol.

[43] Martins LO, Durao P, Brissos V, Lindley PF (2015). Laccases of prokaryotic origin: enzymes at the interface of protein science and protein technology. Cell Mol Life Sci.

[44] Wen X, Zeng Z, Du C (2019). Immobilized laccase on bentonite-derived mesoporous materials for removal of tetracycline. Chemosphere.

[45] Bilal M, Adeel M, Rasheed T (2019). Emerging contaminants of high concern and their enzyme-assisted biodegradation-a review. Environ Int.

[46] Kumar S, Neeraj VK, Karn SK (2018). Biodegradation of phenol by free and immobilized *Candida tropicalis* NPD1401. Afri J Biotech.

[47] Zhang K, Yang W, Liu Y (2020). Laccase immobilized on chitosan-coated Fe_3_O_4_ nanoparticles as reusable biocatalyst for degradation of chlorophenol. J Mol Struct.

[48] Chen Z, Yao J, Ma B, Liu B, Kim J, Li H, Zhu X, Zhao C, Amde M (2021) A robust biocatalyst based on laccase immobilized superparamagnetic Fe 3 O 4@SiO 2-NH 2 nanoparticles and its application for degradation of chlorophenols. Chemosphere 291(Pt 1):132727 10.1016/j.chemosphere.2021.132727 34743799

[49] Brugnari T, Contato AG, Pereira MG et al (2021) Characterisation of free and immobilized laccases from *Ganoderma lucidum*: application on bisphenol a degradation. Biocatalysis Biotransformation 39(1):71–80

[50] Bala JD, Lalung J, Smail N (2014). Biodegradation of palm oil mill effluent (POME) by bacteria. Int J Sci Res.

