# Supplementary material for: 2,4-Dichlorophenol biotransformation using immobilized marine halophilic Bacillus subtilis culture and laccase enzyme: application in wastewater treatment
Source: J Genet Eng Biotechnol. 2022 Sep 16;20:134. doi: 10.1186/s43141-022-00417-1 (PMC9481827; doi:10.1186/s43141-022-00417-1)
Supplement: Supplementary file 1 — Additional file 1: Supplementary Figure 1. Gamma hemolysis of Bacillus subtilis AAK (did not shown any hemolytic activity) against blood in the blood agar medium. [file 43141_2022_417_MOESM1_ESM.docx]

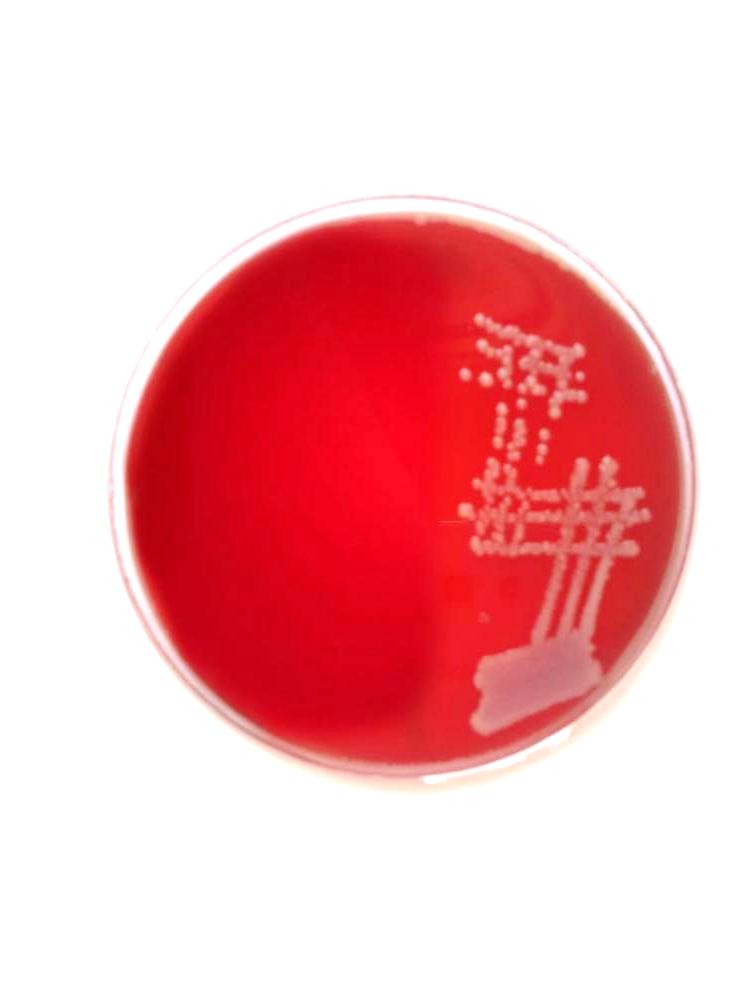


No inoculation

*Bacillus subtilis* AAK

**Figure 1: Gamma hemolysis of *Bacillus subtilis* AAK (did not shown any hemolytic activity) against blood in the blood agar medium**
